# Tail Docking of Piglets 2: Effects of Meloxicam on the Stress Response to Tail Docking

**DOI:** 10.3390/ani10091699

**Published:** 2020-09-20

**Authors:** Rebecca Morrison, Paul Hemsworth

**Affiliations:** 1 Rivalea (Australia), Research and Innovation, Redlands Road, Corowa 2640, Australia; 2 Animal Welfare Science Centre, Faculty of Veterinary and Agricultural Sciences, University of Melbourne, Parkville, Victoria 3010, Australia; phh@unimelb.edu.au

**Keywords:** pigs, tail docking, pigs, tail docking, pain, stress, welfare

## Abstract

**Simple Summary:**

Previous research has shown that tail docking causes an acute stress response and tail docking using the cauterisation procedure may be less aversive than the clipper procedure. This experiment examined the efficacy of meloxicam in mitigating acute stress responses to tail docking. Cauterisation was less aversive than the clipper procedure based on the stress response after docking. Meloxicam mitigated the cortisol response at 30 min after tail docking with the clipper and the behavioural response in the first 60 min after tail docking with the clipper. The commercial viability and implementation of meloxicam requires consideration before it is recommended for use compared to cauterisation alone, as it requires additional handling of piglets and higher costs.

**Abstract:**

This experiment assessed the efficacy of the cauterisation procedure with or without pain relief (injectable meloxicam) in mitigating the acute stress response to tail docking. Male piglets (*n* = 432) were allocated to the following treatments at 2-d post-farrowing: (1) no handling, (2) sham handling, (3) tail docked using clippers, (4) tail docked using a cauteriser, (5) meloxicam + clipper, and (6) meloxicam + cauteriser. Meloxicam treatments used Metacam^®^ at 5 mg/mL injected i.m. 1 h prior to tail docking. Blood samples were collected at 15 and 30 min post-treatment and analysed for total plasma cortisol. Behaviours indicative of pain such as escape attempts, vocalisations and standing with head lowered were measured. The duration of vocalisations and frequency of escape attempts during treatment were greater in all tail docking treatments compared to the sham treatment. Piglets in the clipper treatment had higher (*p* < 0.05) cortisol concentrations at 30 min but not 15 min after treatment and stood for longer (*p* < 0.001) with head lowered in the first 60 min after treatment than those in the cauterisation treatment. Meloxicam reduced (*p* < 0.05) both the cortisol response at 30 min after tail docking with the clipper as well as the behavioural response in the first 60 min after tail docking with the clipper. In comparison to the sham treatment, cortisol concentrations at 15 min were higher in the two tail docking treatments whereas the tail docking treatments with meloxicam were similar to the sham handling treatment. In comparison to the sham handling treatment, cortisol concentrations at 30 min post-docking were higher (*p* < 0.05) only in the clipper treatment. While cauterisation appears to be less aversive than the clipper procedure, the administration of meloxicam did not mitigate the behavioural response during tail docking using either procedure, but reduced standing with head lowered in the first hour after docking for both methods. The commercial viability of administration of meloxicam requires consideration before it is recommended for use compared to cauterisation alone, as it requires additional handling of piglets and costs.

## 1. Introduction

Tail biting is both an economic and welfare problem of pigs that involves destructive chewing of pen-mates’ tails. While the early stages of tail biting are not well documented, it is generally accepted that once the tail is bleeding, possibly with mouthing of the tail by other pigs leading to breaking the skin, the problem of tail biting can rapidly escalate as other pigs are attracted to the tail [1]. Tail biting therefore can result in wounding and bleeding and more severe consequences such as infection, spinal abscess, paralysis, and, in extreme cases, death [2]. The aetiology of tail biting remains poorly understood and potential factors predisposing tail biting are numerous, e.g., crowding, poor ventilation, breakdown in the food or water supply, poor quality diets, and breed type. Despite considerable research, the underlying behavioural mechanisms for tail biting are not well understood. While management and housing factors should be carefully examined in cases of tail biting, tail docking is a common procedure for reducing the risk of tail biting behaviour, and there is evidence that the procedure reduces the numbers of tail-bitten pigs [3,4]. Tail docking of piglets commonly involves the use of either side-cutter pliers (clippers) or a cauterising tail-docking iron (cauterisation) with docking occurring between 1.5 and 2.5 cm from the base of the tail, in between vertebra [5].

There is limited information in the scientific literature on the effects of different procedures of tail docking on pain, particularly in terms of the magnitude and duration of pain, and in fact, whether it is necessary to provide pain relief with these procedures. Our previous research showed that the physiological, behavioural, and neurophysiological responses of pigs to tail docking showed that tail docking of 2-d old piglets using either the clipper or cauterisation procedure caused an acute stress response, and indicated that cauterisation may be less aversive than the clipper procedure [6,7,8].

The need for pain relief to be provided for a painful husbandry procedure that causes an acute stress response remains controversial. Pain relief is required to be used in Canada for tail docking procedures in pigs [5]. There is limited information in the scientific literature assessing practical medication strategies to reduce the acute pain of tail docking. Meloxicam is a non-steroidal anti-inflammatory drug (NSAID) that becomes effective approximately 30–60 min after administration. Meloxicam works by blocking the action of cyclo-oxygenase, which is involved in the production of prostaglandins [9]. Prostaglandins are produced by the body in response to injury and certain diseases and conditions, and cause pain, swelling, and inflammation. Meloxicam blocks the production of prostaglandins and is therefore effective at reducing inflammation and pain [10].

Kells et al. [11] showed that a topical lignocaine cream (EMLA cream—2.5% Lignocaine and 2.5% Prilocaine) applied 60 min prior to tail docking treatment was effective at reducing the neurophysiological (EEG) response to tail docking. In the same experiment, the use of cauterisation also appeared to mitigate this nociceptive response, although to a lesser extent than the topical anaesthetic. The topical anaesthetic cream contained the anaesthetic agents lignocaine and prilocaine, which penetrate the skin and block signals generated by the activation of nociceptors in the dermal and subdermal regions, preventing any generated nociceptive signals from reaching the brain [12]. However, this topical anaesthetic or other medications that are not registered for use in pigs were not investigated in this experiment. The practicality of applying a topical anaesthetic cream to the base of each tail 60 min prior to docking and the possibility of the medication becoming an attractant to other piglets requires examination.

Pain is difficult to study because it is an inherently subjective experience but indirect indicators have been used to study pain in animals. Behavioural indicators that have been used to assess pain in pigs undergoing painful procedures include vocalisation and escape attempts during the procedure, and changing postures such as standing with head lowered [13,14,15]. Pain stimuli cause the release of hormones from the hypothalamo-pituitary adrenal axis and sympatho-adrenal medullary system in mammals and birds and their equivalents in fish [15] and glucocorticoids are generally accepted as a measure of stress [16,17] and have also been used to assess pain in pigs [13,14,15,18,19]. This experiment compared the physiological and behavioural responses of piglets to study the efficacy of meloxicam in mitigating acute stress responses to tail docking.

## 2. Materials and Methods

All animal procedures were conducted with prior institutional ethical approval (Protocol 13B068C) under the requirement of the New South Wales Prevention of Cruelty to Animals Act (1979) in accordance with the National Health and Medical Research Council/Commonwealth Scientific and Industrial Research Organisation/Australian Animal Commission Australian Code of Practice for the Care and Use of Animals for Scientific Purposes.

The experiment was conducted between October and December (spring and early summer) at the Rivalea Australia Research and Innovation unit, Corowa NSW, Australia. Seventy two sows (Large White × Landrace) and their litters were selected. The sows farrowed in individual farrowing crates. Six entire, healthy male pigs were selected from each of the 72 litters when they were approximately 2 d post-birth (432 pigs in total). The pigs were randomly allocated to treatment and pigs were individually identified by a written treatment letter (i.e., A–F) on their back with a black stock marker pen.

The following treatments were imposed:

1. No handling.
2. Sham handling—Piglets were individually held the same way as the other treatments for approximately 30 s, before being returned to their pens.
3. Tail docking using side-cutters (‘Clipper’)—A clean, disinfected side-cutter (clipper) was used to cut approximately 2 cm from the base of the tail in between the second and third vertebrae. A disinfectant was applied to the wound and the piglet was returned to its pen.
4. Tail docking using a Stericut^®^ Tail cauteriser (‘Cauterisation’)—A clean disinfected gas operated Stericut^®^ tail docker was used to cut the tail at the same location as in the clipper treatment. A disinfectant was applied to the wound and the piglet was returned to its pen.
5. Meloxicam-an intramuscular injection of Metacam^®^-5 mg/mL (0.1 mL/1.25 kg pig) 1 h prior to tail docking using clippers.
6. Meloxicam- intramuscular injection of Metacam^®^-5 mg/mL (0.1 mL/1.25 kg pig) 1 h prior to tail docking using cauteriser.

Piglets in the meloxicam treatments were picked up gently and injected with Metacam^®^ 60 min prior to tail docking. All piglets, except those in the no handling treatment, were handled in a similar manner at treatment imposition: they were individually and gently picked up from their farrowing crate and held, supported under the arm of the technician with their hind area exposed, for approximately 30 s before returning them to their farrowing crate.

Piglets in the no handling and sham handling treatment had their tails removed after blood sampling and behavioural observations were completed as recent sporadic and unpredictable occurrence of tail biting in commercial herds in the region had occurred. Therefore, the data for growth performance of piglets in these two treatments were not included in the analysis.

### 2.1. Stress Physiology

Blood samples were collected by jugular venipuncture. The blood samples were taken at 15 and 30 min post-tail docking. The blood sampling was conducted by trained personnel who were able to obtain a blood sample within 20 s of the piglet being picked up. The blood was collected into 2 mL Vacutainer tubes (BD, Franklin Lakes, NJ, USA) treated with lithium heparin and stored on ice. The individual samples were centrifuged at 7000 rpm and the plasma was poured off and stored frozen at −20 °C until analysed. The samples were assayed for total cortisol at the University of Western Australia. Plasma concentrations of cortisol were measured in duplicate by radioimmunoassay using Immuchem^TM^ Coated Tube Cortisol RIA kits (MP Biomedicals, Belgium). The limit of detection was 0.2 µg/dL. Quality control samples (7.1 and 25.2 µg/dL) were used to estimate inter- (6.4% and 3.8%) and intra-assay (7.4% and 4.8%) coefficients of variation.

### 2.2. Behaviour

During the treatment, an escape attempt was defined as a body movement carried out to effect an escape (i.e., rapid leg thrust while being held by the technician) as described by Marchant-Forde [18]. The duration of vocalisations was recorded during treatment, from the time piglets were picked up to when they were placed back in the pen after treatment. The behaviour of the six treatment pigs in each litter was videotaped by using mounted cameras (HD Sports cameras) that enabled view of the whole farrowing crate. The piglet behaviours observed are described in Table 1 and the duration of these behaviours were sampled as follows. Each of the six piglets per litter was observed continuously for the first 60 s every 5 min in the first 60 min post-treatment. This resulted in six sets of 60-s observations on each of the six piglets in each litter (i.e., a total of 12 min per piglet observed in the first 60 min post-treatment).

### 2.3. Growth Performance and the Number of Piglets That Died Due to Illness, were Euthanised, or Were Removed Due to Illness

The piglets were weighed individually immediately prior to the treatment and then at 7 d post-treatment and at weaning (average of 26 d of age). The total number of piglets that died due to illness, were euthanised, or were removed due to illness was recorded.

### 2.4. Statistical Analysis

Statistical analyses were performed using SPSS (Version 21-SPSS Inc., Chicago, IL, USA). All data were analysed for normality and transformed where appropriate. An analysis was conducted using the univariate general linear model, using each piglet as the experimental unit and the sow as the random factor. When significant treatment differences (*p* < 0.05) were detected, least significant difference (LSD) tests were used for pairwise comparisons between all treatments. Chi-squared analysis was used to analyse treatment effects on the total number of piglets that died due to illness, were euthanised or were removed due to illness.

## 3. Results

### 3.1. Mortality

There was no significant difference (X^2^ = 3.68; *p* = 0.505) between the total number of piglets that died or were removed due to illness and injury between treatments. There was a trend for more deaths when clippers with or without meloxicam were used compared to cauterisation with or without meloxicam (13.2% and 6.9% mortality post-treatment, respectively, X^2^ = 3.11, *p* = 0.078).

### 3.2. Cortisol Concentrations

There were treatment effects on cortisol concentrations at 15 and 30 min post-treatment (*p* < 0.001; Table 2). In comparison to the sham treatment, cortisol concentrations at 15 min post-treatment were higher (*p* < 0.05) in the clipper and cauterisation treatment. Both tail docking treatments with meloxicam were similar to the sham treatment (*p* > 0.05). Cortisol concentrations in the no handling treatment were lower than in the other four treatments (Table 2).

Although cortisol concentrations at 30 min post-treatment in both tail docking treatments were similar to the sham handling treatment (*p* > 0.05), cortisol concentration was higher (*p* < 0.05) in the clipper than the cauterisation treatment (Table 2). However, cortisol concentrations at 30 min post-treatment in the sham handling treatment and both tail docking procedures with meloxicam were similar (*p* > 0.05). Furthermore, all treatments apart from the cauterisation and meloxicam treatment had higher cortisol concentrations at 30 min post-treatment than the no handling treatment.

### 3.3. Behaviour

Scooting, scratching, shivering, playing, and frolicking were not observed during the observation period. There were significantly more (*p* < 0.05) vocalisations and escape attempts in all tail docking treatments compared to the sham handling treatment (Table 3). Piglets in the clipper treatment spent more time (*p* < 0.05) standing with their head lowered compared to other treatments. There was no significant difference (*p* > 0.05) between treatments in other behaviours observed in the 60-min period post docking.

### 3.4. Growth Performance

There was no significant difference (*p* > 0.05) in weaning weight or rate of gain between the clipper, cauterisation, clipper and meloxicam, and cauteriser and meloxicam treatments (Table 4).

## 4. Discussion

This experiment used a broad examination of physiological and behavioural responses of piglets to examine the stress responses of piglets to tail docking with the use of meloxicam. Tail docking using the clippers or cauterisation elicited a greater cortisol response at 15 min post-treatment compared to the sham handling treatment. At 30 min after treatment, cortisol concentrations in both tail docking treatments and the sham treatment were similar, however, the cauteriser treatment elicited a lower cortisol response than the clipper treatment. These results are similar to those of our previous research [6] and Sutherland et al. [8], although the pigs in the latter study were considerably older (6 d rather than 2 d of age). Prunier et al. [19] also found that cortisol concentrations did not differ between tail docking using cauterisation and handling alone treatments for up to 180 min post-tail docking, providing further evidence that cauterisation is less aversive than the use of clippers to tail dock. However, Marchant-Forde et al. [18] found no difference in cortisol concentration 45 min post-tail docking using clippers or cauterisation and speculated that cortisol had peaked and returned to baseline levels prior to 45 min post-treatment.

In comparison to the sham handling treatment, meloxicam reduced the cortisol response at 15 min post-treatment to the two tail docking procedures. However, while tail docking with clippers was higher at 30 min post-treatment than tail docking with cauterisation, meloxicam reduced the magnitude of the cortisol response at 30 min in tail docking with clippers to a similar level as that found in tail docking with cauterisation with or without meloxicam. Furthermore, while vocalisation and escape attempts at the time of docking in the two tail docking procedures with or without meloxicam were unaffected, standing with the head lowered was higher in the 60 min after docking in tail docking with clippers than tail docking with cauterisation with or without meloxicam and tail docking with clippers with meloxicam. Head being lowered has been previously suggested to be an indicator of pain in castrated piglets [13]. These cortisol and behavioural data in the present study indicate that meloxicam reduces the stress response with these two tail docking procedures, particularly in the more stressful procedure of docking with clippers. Meloxicam, like other non-steroidal anti-inflammatory drugs, is believed to exert anti-nociceptive effects mainly through inhibition of peripheral inflammatory responses and there is some evidence that it may also have central and pre-emptive analgesic effects [10].

Tail docking using clippers or cauterisation caused an increase in the duration of piglet vocalisations and number of escape attempts during the tail docking procedure and these behaviours were not mitigated by the use of meloxicam, which is in agreement with Tenbergen et al. [21]. Meloxicam used in other domestic species has been shown to mitigate pain-associated behaviour, such as less social isolation and more locomotion, as well as reducing the cortisol response post-treatment [1,21,22,23]. However, meloxicam does not appear to be effective at blocking the acute pain associated with the surgery itself, based on neurophysiological analysis by Kells et al. [11] in which the administration of meloxicam 60 min prior to tail docking did not affect the nociceptive response during the tail docking surgery. Marchant-Forde et al. [18] showed that piglets that had their tail docked by cauterisation emitted more squeals per s with higher mean and peak frequencies compared to tail docking with clippers. The authors state that the cauterisation procedure took 20% longer than that of clippers, thereby exposing the piglets to longer handling. In the present experiment, and research conducted previously [6], piglets were all held for the same amount of time to ensure that there were no confounding factors involved.

The effects of the injection per se on the stress response was not assessed in this experiment, however McGlone et al. [24] found that a single intramuscular injection given to 3–5 d old piglets (and grower pigs) did not affect their behaviour during a 60 min period after injection or cortisol concentrations at 60 min after injection. The use of alternative pathways to deliver pain relief to piglets has been investigated. For example, Bates et al. [25] showed a reduction in pain indicators post-tail docking following trans-mammary delivery of meloxicam. However, the authors concluded that even though meloxicam was transferred from sows to piglets through milk, the concentrations found in piglets were much lower than that injected in sows, indicating further research required to refine the dose.

Herskin et al. [26] reported that behavioural indicators of pain were reduced when lidocaine was used for local anaesthesia and that pre-emptive administration of meloxicam did not alleviate procedural pain. The study also concluded that neither a local anaesthetic (lidocaine) nor a NSAID (meloxicam) fully eliminated pain during or after tail docking in piglets and that further research is needed in order to develop a more reliable protocol for pain relief. Indeed, it may in fact be unrealistic to remove all pain associated with tail docking, and as such the aim may need to be to minimise pain associated with the procedure. Furthermore, future research should be obviously directed on housing and management systems to reduce the need for tail docking of piglets in the first instance.

There were no treatment effects on the number of piglets that died or were removed due to illness and injury and growth indicating that administration of meloxicam prior to tail docking did not provide any longer-term benefits to the piglets in terms of health, weight gain, and survival. These data provide further evidence that tail docking causes an acute stress response, which does not impact on the biological fitness of the animal [6] and the results are in agreement with other reports that found no relationship between pain relief and weight gain of piglets [21,27]. Since piglets in the no handling and sham handling treatments were tailed docked immediately after the behaviour and cortisol measurements were taken (60 min post-treatment) to avoid tail biting, it was not possible in the present experiment to compare the growth performance of tail docked pigs to those with intact tails because of the increased risk of tail biting.

While not statistically significant, it is noteworthy that there was a trend for fewer piglets that died due to illness, were euthanised, or were removed due to illness in the cauterisation treatment. This tail docking procedure involves the burning of the tail tissue and searing the wound, which may reduce the risk for bacteria to gain entry via the tail wound post-tail docking. In contrast, tail docking with clippers leaves an open wound [28]. This requires further investigation involving larger numbers of animals, since there may be a long-term welfare benefit in terms of growth and survival of pigs docked using the cauterisation.

## 5. Conclusions

Tail docking of piglets by either clipper or cauterisation caused an acute stress response based on behaviour during treatment and behaviour and cortisol during 30–60 min period post-treatment. Cauterisation appeared to be less aversive based on this acute stress response at 30 min post-treatment. Meloxicam reduced the stress response with these two tail docking procedures, particularly with the procedure of docking with clippers. The commercial viability of administration of meloxicam requires consideration before it is recommended for use compared to cauterisation alone as it requires additional handling of piglets prior to tail docking and higher costs.

## Figures and Tables

**Table 1:** Description of piglet behaviours observed (modified from Hay et al. and Hurnik et al. [13,20].

Behaviours	Description
Standing	Upright position with bodyweight supported by all four legs.
Standing with head lowered	Upright position with bodyweight supported by all four legs. Head lower than shoulders.
Sitting	Body weight supported by the hind-quarters and front legs.
Lying (with sow contact)	Maintaining a recumbent position in contact with a part of the sow.
Lying (without sow contact)	Maintaining a recumbent position not in contact with a part of the sow.
Idle	Not performing any behaviour
Walking	Relatively low speed locomotion in which propulsive force derives from action of legs.
Massaging udder/Nursing	Nose in contact with the udder and/or teat in mouth-assumed to be suckling.
Asleep	Eyes closed while lying down.
Playing/frolicking	Head shaking, springing (sudden jump or leap), running with horizontal and vertical bounces.
Scooting	Causal part of body being dragged across ground.
Scratching	Scratching the rump against the floor or walls of the pen.
Shivering	Shivering as with cold.

**Table 2:** Effect of treatment on mean total cortisol concentrations at 15 and 30 min post-treatment.

	No Handling	Sham	Clipper	Cauterisation	Clipper + Meloxicam	Cauterisation + Meloxicam	SEM *	*p* Value
Cortisol (ng/mL)								
15 min	88.6 ^a^	138.4 ^b^	186.7 ^c^	169.5 ^c,d^	163.2 ^b,c,d^	144.3 ^b,d^	3.97	0.000
30 min	212.6 ^a^	276.2 ^b,c^	317.7 ^b^	267.5 ^c^	261.8 ^c^	238.7 ^a,c^	6.73	0.001

* Standard error of mean; ^a,b,c,d^ Within rows values with different superscripts are significantly different (*p <* 0.05; Fisher’s LSD test).

**Table 3:** Effect of treatment on the duration of vocalisations and number of escape attempts during imposition of treatments and duration of behaviours of piglets for the first 60 min after treatment.

Variable *	No Handling	Sham	Clipper	Cauterisation	Clipper + Meloxicam	Cauterisation + Meloxicam	SEM	*p* Value
*At treatment imposition* Duration of vocalisations during treatment (s)	-	1.2 ^a^ (1.4)	2.1 ^b^ (4.4)	1.9 ^b^ (3.6)	2.0 ^b^ (4.0)	1.9 ^b^ (3.6)	0.04	0.000
Number of escape attempts during treatment	-	0.9 ^a^ (0.8)	1.8 ^b^ (3.2)	1.8 ^b^ (3.2)	1.9 ^b^ (3.6)	1.7 ^b^ (2.9)	0.04	0.000
*First 60 min after treatment* Standing (normal)	10.2 (104.0)	11.8 (139.2)	11.8 (139.2)	10.4 (108.2)	14.8 (219.0)	15.5 (240.3)	0.19	0.149
Standing (head lowered)	4.2 ^a^ (17.6)	4.1 ^a^ (16.8)	10.7 ^b^ (115.5)	5.2 ^a^ (27.0)	4.2 ^a^ (17.6)	5.8 ^a^ (33.6)	0.15	0.000
Sitting	6.0 (36)	6.2 (38.4)	3.7 (13.7)	6.8 (46.2)	6.1 (37.2)	6.5 (42.3)	0.33	0.686
Lying (with sow contact)	11.1 (123.2)	13.5 (182.3)	6.1 (37.2)	13.0 (169)	10.3 (106.1)	14.0 (196.0)	0.35	0.056
Lying (without sow contact)	20.1 (404.0)	20.8 (432.6)	17.9 (320.4)	21.4 (457.9)	18.0 (324.0)	18.1 (327.6)	0.30	0.875
Idle	7.3 (53.3)	6.9 (47.6)	8.5 (72.3)	7.2 (51.8)	7.6 (57.8)	8.1 (65.6)	0.15	0.965
Walking	6.8 (46.2)	6.3 (39.7)	11.5 (132.3)	7.6 (57.8)	9.2 (84.6)	8.8 (77.4)	0.16	0.134
Massaging udder/Nursing	11.5 (132.3)	10.6 (112.4)	8.7 (75.7)	7.6 (57.8)	13.6 (185.0)	13.6 (185.0)	0.27	0.347
Asleep	20.3 (412.1)	21.3 (453.7)	18.4 (338.6)	21.5 (462.3)	18.4 (338.6)	18.3 (334.9)	0.21	0.667

^a,b^ Within rows values with different superscripts are significantly different (*p* < 0.05); * All variables were square root transformed prior to statistical analysis. Transformed means are presented and back transformed means presented in parentheses.

**Table 4:** Effect of treatment on growth performance of piglets.

Variable	Clipper	Cauterisation	Clipper + Meloxicam	Cauterisation + Meloxicam	SEM	*p* Value
Live weight prior to treatment (kg)	1.90	1.96	1.95	1.97	0.025	0.797
Weaning weight (kg) *	6.34	6.33	6.33	6.31	0.120	1.00
Growth rate treatment to weaning (g/day) *	0.209	0.207	0.206	0.207	0.005	0.997

* Data not included for no handling and sham handling treatments as these piglets were tail docked immediately after the behaviour and physiology measures were taken. Individual weight prior to treatment was used as a covariate in analysis.
